# DIAGNOSTIC VALUE OF THE REACTION AT THE BACILLUS CALMETTE-GUÉRIN VACCINATION SITE IN KAWASAKI DISEASE

**DOI:** 10.1590/1984-0462/2021/39/2019338

**Published:** 2020-08-28

**Authors:** Lilian Martins Oliveira Diniz, Raquel Gomes Castanheira, Yala Gramigna Giampietro, Matheus Sewastjanow Silva, Flávia Duarte Nogueira, Priscila Duarte Pessoa, Thamires Marx da Silva Santos, Gislene Soares Coutinho, Roberta Maia de Castro Romanelli

**Affiliations:** a Universidade Federal de Minas Gerais, Belo Horizonte, MG, Brazil.; b Universidade José do Rosário Vellano, Belo Horizonte, MG, Brazil.

**Keywords:** Vaccines, Kawasaki disease, Bacillus Calmette Guerin vaccine, Vacinas, Síndrome de linfonodos mucocutâneos, Vacina BCG

## Abstract

**Objective:**

To describe the case of an infant - diagnosed with incomplete Kawasaki disease - who developed BCG scar reactivation.

**Case description:**

A 6-month-old patient was admitted to hospital with fever associated with ocular hyperemia, cervical lymphadenopathy, and hyperemic lips, and remained hospitalized for 12 days. The physical examination revealed an inflammatory reaction at the site of the BCG scar, leading to the diagnosis of incomplete Kawasaki disease. The patient was treated with venous immunoglobulin, but presented recurrence of Kawasaki disease, with subsequent onset of coronary artery disease.

**Comments:**

BCG scar reactivation is an important finding in countries where the vaccine is routinely given and may be a useful marker for early diagnosis of Kawasaki disease, especially in its incomplete form.

## RESUMO

**Objetivo:**

Descrever o caso de um lactente - com diagnóstico de Doença de Kawasaki incompleta - que desenvolveu reativação da cicatriz da vacina BCG.

**Descrição do caso:**

Um paciente de 6 meses de idade foi admitido no hospital com febre, associada à hiperemia ocular, linfadenomegalia cervical e fissuras labiais, permanecendo hospitalizado por 12 dias. Apresentava, no exame físico, reação inflamatória no local da cicatriz da vacina BCG, tendo sido feito o diagnóstico de Kawasaki incompleto. O paciente foi tratado com imunoglobulina venosa, mas apresentou recorrência da doença, com posterior surgimento de coronariopatia.

**Comentários:**

A reativação da BCG é um achado importante na doença de Kawasaki em países onde a vacina é aplicada de forma rotineira e pode ser um marcador útil para o diagnóstico precoce da doença de Kawasaki, principalmente em sua forma incompleta.

## INTRODUCTION

Kawasaki disease (KD) is an acute self-limited systemic vasculitis of unknown etiology that predominantly affects children aged <5 years.^1^ ^,^ ^2^ It was first described in Japan by Tomisaku Kawasaki, in 1967, and has been reported worldwide, with the highest rates found in Asian countries.^2^ Its incidence ranges from 265/100,000 children aged <5 years in Japan to 19/100,000 in the United States.^1^ In Brazil, as well as in other Latin American countries, the incidence of the disease has not been reported yet.

 Clinically, KD is characterized by fever lasting ≥5 days associated with at least 4 of the following signs: polymorphic light eruption, bilateral conjunctivitis, erythema and cracking of the lips or oral cavity, edema and skin peeling of the fingers, and cervical lymphadenopathy.^1^ The vasculitis observed in KD patients can cause inflammation of coronary arteries, as well as necrosis and fibrosis of the arterial wall.^1^ The main complication of cardiac involvement is the development of coronary artery aneurysms, fistula, dilatation, and myocardial infarction, which occur as a result of KD in 15 to 25% of untreated children.^1^ Cardiovascular manifestations can be prominent during the acute phase and are an important cause of long-term morbidity and mortality.^1^ ^,^ ^2^ Despite its self-limiting nature, KD requires early diagnosis and prompt treatment to prevent cardiovascular complications.^1^

In the absence of specific diagnostic tests, the detection of KD is based on the assessment of its clinical manifestations and the exclusion of other febrile illnesses.^2^ Incomplete KD should be considered among the differential diagnoses in any infant or child presenting prolonged unexplained fever, <4 of the aforementioned principal clinical findings, and laboratory and/or echocardiographic results consistent with KD.^1^ ^,^ ^2^ ^,^ ^3^ Patients with incomplete KD may experience significant delays in diagnosis.^3^

A major obstacle to diagnosing KD is the lack of gold standards to identify the condition.^4^ ^,^ ^5^ Unfortunately, the current definition of KD, based on the diagnostic criteria, overlaps with other diseases. An important clinical sign not included in the diagnostic criteria is the involvement of erythema and induration at the Bacillus Calmette-Guérin (BCG) scar, which the Japanese literature highlighted as a specific and early sign of KD.^4^ ^,^ ^6^ Acute inflammation at the BCG inoculation site is one of the few features of KD among infants in countries where vaccination against tuberculosis is widely used.^3^ ^,^ ^4^

The first description of BCG scar reaction after KD occurred in 1982.^2^ A literature review conducted by Rezai et al., in 2014, showed that a total of 15 studies reported BCG scar reactivation in KD patients, and 9 of them were case reports.^6^ In Eastern Asian countries, such as Japan, Korea, and Taiwan, almost 50% of KD patients present BCG site inflammation.^4^ As for Latin America, a few cases of BCG scar reactivation have been reported in Costa Rica and Mexico, where BCG scar reaction was present in 15 and 24% of the patients, respectively. Only 18% of them presented incomplete disease.^2^

BCG vaccination site involvement may facilitate prompt diagnosis and prevention of coronary aneurysms, which are the most severe side effects of the disease, especially in patients who do not fulfill the classic criteria of at least four of the five findings, as observed in incomplete KD.^4^ ^,^ ^5^ There are no descriptions of BCG scar reactivation in Brazilian children diagnosed with KD. This study aimed to describe the case of an infant in Brazil - diagnosed with incomplete KD - who had BCG scar reactivation, as well as demonstrate how BCG scar erythema was a useful diagnostic criterion for the disease.

## CASE REPORT

**Figure 1.**
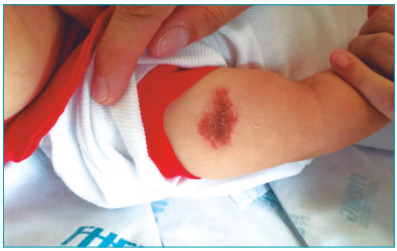
BCG scar reactivation at admission.

A 6-month-old male infant with a history of fever for 12 days was admitted to our 150-bed freestanding children’s hospital in Southeastern Brazil for investigation of the persistent condition. Laboratory tests performed on the 11^th^ day of fever showed that the infant presented anemia, leukocytosis, thrombocytosis, and elevated C-reactive protein (CRP) (Table 1). Upon admission, we observed irritability, markedly hyperemic lips, bilateral conjunctival hyperemia, bilaterally enlarged anterior cervical lymph nodes, without edema of his hands and feet, and without rash. His mother reported that, on the 4^th^ day of fever, she had noticed hyperemia, edema, and induration at the BCG scar, followed by desquamation. At the time of admission, the infant’s lesion measured 3 cm in diameter and was painless to palpation (Figure 1). Based on clinical and laboratory presentation, incomplete KD was suspected, and the administration of intravenous immunoglobulin (IVIG) (2 g/kg) and acetylsalicylic acid (80 mg/kg) was initiated on the 13^th^ day of illness. Two days later, the patient presented a resolution of symptoms: fever remission, resolution of ocular hyperemia and lips erythema, and reduced lymphadenopathy. Lamellar desquamation of fingers and toes occurred on the 18^th^ day of the disease. BCG scar reactivation persisted. An echocardiogram performed on the 18^th^ day of the illness revealed small coronary artery aneurysms measuring 4 and 3.5 mm on the right and left side, respectively. The dose of acetylsalicylic acid was reduced to 5 mg/kg after 3 days of treatment, and the patient was discharged on the 19^th^ day of the disease, after 12 days of hospitalization.

**Table 1:** Laboratory tests according to the day of fever.

	11^th^ day of fever	47^th^ day of fever
Hemoglobin	7.8 g/dL	9.4 g/dL
Leukocyte count	17,590 cells/mm^3^	21,300 cells/mm^3^
Segmented neutrophils	46.5%	57%
Lymphocytes	37.9%	33%
Bands	-	4%
Basophils	0.8%	
Monocytes	9.8%	1%
Eosinophils	2%	5%
Platelet count	998,000 cells/mm^3^	949,000 cells/mm^3^
CRP		239.9 mg/L
AST(SGOT)	25 U/L	22 U/L
ALT(SGPT)	18 U/L	27 U/L

CRP: C-reactive protein; AST (SGOT): aspartate aminotransferase; ALT (SGPT): alanine aminotransferase.

He was readmitted to the Emergency Department on the 47^th^ day of the disease with persistent fever for eight days, showing recurrence of conjunctival hyperemia, lips erythema, edematous feet, and desquamation of his fingertips. The BCG scar still showed signs of mild inflammation, but with a significant improvement since the previous hospitalization. Laboratory tests conducted on the same day detected persistence of anemia, leukocytosis, thrombocytosis, besides elevated C-reactive protein (CRP) (Table 1). An echocardiogram carried out one day later presented right and left coronary artery aneurysms measuring 3.2 and 4 mm, respectively. IVIG was prescribed again on the 49^th^ of the disease, and the acetylsalicylic acid dose was increased to 80 mg/kg. The patient showed good response to treatment 48 hours after the IVIG infusion and was discharged on the 55^th^ day of illness, with the improvement of all symptoms and resolution of erythema and induration at the BCG scar.

## DISCUSSION

KD diagnosis remains a clinical challenge. The lack of a diagnostic test means that its detection depends on an assessment of the presence of specific disease criteria, which, unfortunately, do not identify the illness in all children. Erythema and induration at a previous BCG inoculation site have been described in children with KD who were born in countries where this vaccine is widely administered and is a useful marker for early diagnosis of this disease.^6^ Although its etiology remains unclear, some studies propose that the erythematous changes associated with this reaction are part of a generalized activation of the immune system. A cross-reaction between a possible infectious agent involved in the disease and mycobacterial BCG antigens might be present, contributing to the inflammatory process.^2^ A hypothesis suggests that this cross-reactivity occurs between specific epitopes from the mycobacterial protein 65kD and its homologous human protein.^2^ ^,^ ^3^

BCG scar reactivation in KD patients is significantly more common than in those with other febrile diseases.^4^ Erythema, ulceration, and crusting are usually found at the vaccination site, as seen in this case.^5^ Some reports have shown that fever subsides, and erythema at the BCG scar disappears a few days after immunoglobulin therapy.^6^ The resolution of inflammation at the BCG site could not be observed in the patient studied after the first treatment with IVIG and was only described on the 55^th^ day of illness, after the second dose of IVIG.

The proportion of male patients with erythema at the BCG scar is higher than that of females with the same disease. Also, it seems to be more frequent in younger patients, especially those under 6 months of age.^2^ ^,^ ^6^ Some studies have revealed that more than 70% of the patients with BCG scar reaction are aged 3 to 20 months,^6^ especially between six to 12 months.^4^ There are suggestions that, after BCG vaccination, the prevalence of reaction decreases with time. Our report identified a similar finding concerning the age of the patient presented.

Uehara et al. have also investigated the relationship between the day of hospitalization and the onset of BCG site inflammation. They found that this sign appears in the early stage of the disease: between one and four days after the start of fever.^4^ Our patient presented erythema at the BCG scar on the 4^th^ day of the disease, corroborating what was previously reported.

Despite its diagnostic utility, BCG scar reactivation is not an essential component of the classic KD diagnostic criteria, particularly among patients who do not meet the criteria for complete KD. Some studies have shown that BCG site inflammation is more prevalent in KD patients than cervical lymphadenopathy and rash, demonstrating, thus, that it can be a useful criterion in the detection of incomplete cases of the disease, as in the patient described herein.^4^ ^,^ ^6^ Prevalence of cervical lymphadenopathy is lower in young KD patients than in older ones. Therefore, a BCG scar reaction in younger KD patients would be a more useful sign than cervical lymphadenopathy for diagnosing the disease in this age group.^2^

Although laboratory test results are nonspecific, they provide supporting evidence for the KD diagnosis. Laboratory test results during the acute phase typically reveal normal or high white blood cell counts and elevated CRP. Anemia occurs frequently and resolves with the end of inflammation. Thrombocytosis is a characteristic of KD; however, it usually does not appear until the 2^nd^ week of the illness.^1^ These changes demonstrate an acute inflammatory process and intense immune activation of the cytokine cascade.^1^ KD patients with BCG scar reactivation tend to show marked leukocytosis and thrombocytosis associated with increased inflammatory markers, as seen in this case.^4^

Recurrence of KD is uncommon and observed in only approximately 3% of patients. It is more common among children aged <3 years at the first episode and may occur within eight years after the first episode. The patient studied presented early recurrence of symptoms, which manifested about 40 days after the onset of fever, and 30 days after IVIG treatment. Recurrence is more widely documented in Japanese literature, but its risk factors remain unclear.^1^ So far, BCG scar reactivation has not been described in cases of KD recurrence.

Japanese guidelines classify coronary artery aneurysms based on the absolute or relative internal luminal diameter: small aneurysms are defined as localized dilatations with an internal luminal diameter <4 mm, medium aneurysms present >4 and <8 mm, and large or giant aneurysms have >8 mm.^1^ ^,^ ^2^ The goal of therapy is to reduce inflammation and arterial damage and prevent thrombosis. In our patient, late treatment after the 10^th^ day of illness was associated with mild coronary artery dilatation. A literature review^6^ showed that 10.3% of patients with BCG scar reactivation presented coronary artery abnormalities. Some authors suggest that there is no association between BCG scar reactivation and coronary artery lesions. They declare that these findings are not useful for predicting the presence of coronary aneurysms.^2^ ^,^ ^4^ ^,^ ^6^

Tuberculosis remains endemic throughout Brazil, where a permanent universal vaccination program for BCG has been adopted for infants. In countries where BCG vaccination is recommended, its scar site reaction should be considered a useful marker for early KD diagnosis to prevent cardiovascular complications.
